# Health Literacy and Diabetes Knowledge: A Nationwide Survey in a Multi-Ethnic Population

**DOI:** 10.3390/ijerph18179316

**Published:** 2021-09-03

**Authors:** P. V. Asharani, Jue Hua Lau, Kumarasan Roystonn, Fiona Devi, Wang Peizhi, Saleha Shafie, Sherilyn Chang, Anitha Jeyagurunathan, Chua Boon Yiang, Edimansyah Abdin, Janhavi Ajit Vaingankar, Chee Fang Sum, Eng Sing Lee, Rob Van Dam, Siow Ann Chong, Mythily Subramaniam

**Affiliations:** 1 Research Division, Institute of Mental Health, Singapore 539747, Singapore; Jue_Hua_LAU@imh.com.sg (J.H.L.); K_ROYSTONN@imh.com.sg (K.R.); Fiona_Devi_SIVA_KUMAR@imh.com.sg (F.D.); peizhi_wang@imh.com.sg (W.P.); saleha_shafie@imh.com.sg (S.S.); Sherilyn_Sh_CHANG@imh.com.sg (S.C.); anitha_jeyagurunathan@imh.com.sg (A.J.); boon_yiang_chua@imh.com.sg (C.B.Y.); edimansyah_abdin@imh.com.sg (E.A.); janhavi_vaingankar@imh.com.sg (J.A.V.); siow_ann_chong@imh.com.sg (S.A.C.); mythily@imh.com.sg (M.S.); 2 Admiralty Medical Centre, Khoo Teck Puat Hospital, Singapore 730676, Singapore; sum.chee.fang@ktph.com.sg; 3 Clinical Research Unit, National Healthcare Group Polyclinics 3 Fusionopolis Link, Singapore 138543, Singapore; eng_sing_lee@nhgp.com.sg; 4 Saw Swee Hock School of Public Health, National University of Singapore, Singapore 117549, Singapore; rob.van.dam@nus.edu.sg

**Keywords:** health literacy, diabetes knowledge, lifestyle, chronic diseases, physical activity

## Abstract

Health literacy is a key determinant of the public health and health climate of the nation. This study examined the functional health literacy of the nation, factors associated with health literacy, and its relationship with diabetes recognition. This cross-sectional survey recruited participants (N = 2895) who were 18 years and above from a population registry through disproportionate stratified sampling. The Brief Health Literacy Screen and other questionnaires were administered through face-to-face interviews, in one of the four national languages (English, Chinese, Malay or Tamil). The majority (80.5%) had adequate functional health literacy and were able to recognise symptoms of diabetes correctly (83.5% overall; 83.7% and 82.2% in those with adequate and inadequate health literacy, respectively). Those with inadequate health literacy had a higher incidence of chronic conditions (*p* < 0.001) compared to those with adequate health literacy in bivariate analysis. The majority of the sample had sufficient levels of physical activity (83.3%), and more than half reported an unhealthy lifestyle (57.4%). Older age, Chinese ethnicity, those who were employed, with lower education (secondary or below), and were married had significantly higher odds of inadequate health literacy. Health literacy was not associated with lifestyle, physical activity, chronic conditions and diabetes recognition. Health literacy interventions should focus on the disadvantaged social groups for improving their health literacy.

## 1. Introduction

Health literacy (HL) refers to the cognitive and social skills that equip an individual to receive, analyse and understand health information in order to make appropriate health care decisions and lifestyle choices [1]. The skills related to HL are grouped under three main categories of functional (basic numeracy, skills to read, write and gather health-related information in health care milieu), interactive (higher cognitive skills to gather, comprehend and apply necessary information to changing contexts to make effective decisions), and critical HL (skills to analyse and make use of the information to improve health) [2,3]. Having adequate HL empowers an individual to engage in decision making and utilise the advantages of health care services. Those with adequate HL take better care of their health and have better treatment outcomes, thus contributing towards improvements in public health [4,5]. A meta-analysis of studies further confirmed that adequate HL is significantly associated with better disease outcomes of diabetes (glycemic control, knowledge and self-care) [6]. Thus, achieving higher HL is an essential goal for the prevention and management of chronic conditions [7].

In contrast, those with inadequate HL often have difficulty reading prescriptions, analysing the risks of medical procedures they are undergoing and understanding or following instructions given by the care providers [8]. This will eventually lead to poorer care coordination and dissatisfaction among both parties, leading to treatment dropouts [8]. Conversely, inadequate HL is associated with a higher incidence of chronic diseases and healthcare utilisation, as well as lower disease knowledge, self-care, and medication adherence [9,10,11]. Singh et al. [12] reported that those with inadequate HL were less likely to understand the prescription information which interfered with their self-management of the disease. Thus, HL not only enhances health care outcomes and service efficiency, but also promotes equality of health.

The HL of the population is a determinant of the health care climate of the nation and is central to understanding health disparities, which can enable timely interventions through health promotion activities. A higher prevalence of adequate HL in a population is also a measure of the efficiency of the awareness/educational initiatives implemented by the health authorities [3,13]. A low proportion of adequate HL within a population is associated with a higher incidence and severity of diseases and adverse health outcomes such as mortality and frequent readmissions, resulting in a higher utilisation of resources [3,13]. The European HL survey showed that nearly 47% of the respondents [14] had inadequate HL. A population level study in the Netherlands among those with chronic conditions measured all three domains of HL using a four-point scale and reported higher scores (scores 3 and 4) for functional (84%) and interactive (83%), but not for critical HL (52%) [15]. The study also showed that older age, lower education, having multimorbidity and lower income were associated with inadequate HL [15]. The National America’s Adults Literacy survey (NAAL) in 2003 showed that only 12% of the participants scored a ‘proficient’ level in the HL scale, while 53% had intermediate scores, and 22% and 14% had basic or below basic scores, respectively [16]. The survey also showed that only 32% of adults had health numeracy. A similar national level survey in Israel reported that 69% of the sample had adequate HL, while the rest had either problematic or inadequate HL [17]. Age, education, language barriers, low literacy levels, and existing medical conditions were shown to be related to inadequate HL [18]. Another study that employed the Brief Health Literacy Screen (BHLS) reported a HL of 74.8% in residents of Lagos, Nigeria [19]. The HL of the participants varied across studies and between countries based on the domain of HL measured (functional, interactive or critical) and sociodemographic characteristics of the participants. A study in Korea looked at functional HL [20], while the European HL survey and the population study in the Netherlands measured all three domains of HL [15,21]. Given the importance of this construct in public health, there is an urgent need to understand the HL of the population for better planning of health policies in view of the rising prevalence of chronic conditions globally.

Diabetes is a chronic condition with a high prevalence and is a major health problem globally. It is one of the top 10 causes of mortality and morbidity worldwide [22]. HL empowers patients with diabetes with knowledge that helps them to recognise symptoms of diabetes, thus aiding in the prevention and management of the disease [23]. Failure to recognise these symptoms delays timely medical interventions and leads to further complications. A recent review that synthesised the data from randomised controlled trials concluded that HL improves diabetes knowledge, self-efficiency and quality of life [13]. Thus, HL is an indicator of how well individuals recognise and manage their health conditions.

To date, no studies have been conducted in Singapore to assess the HL of the population. Ko et al. [24] studied HL in a convenient sample of hypertensive patients and showed that 45.1% of the sample had adequate HL. Another study reported that 56.3% of a clinical sample with rheumatic disease had adequate HL [25]. As with the previous global studies on HL [17,18,19,20], both the studies employed different assessment tools for capturing HL (Short Test of Functional Health Literacy and Rapid Estimation of Adult Literacy in Medicine), thus making comparisons across the studies difficult. Another study from Singapore used a subscale (four items) from the Health Literacy Questionnaire to capture the relationship between various domains of HL (finding information, appraising, understanding to act and managing their health) with the mode of health information seeking [26]. The study concluded that the domains for appraising health information and e-health literacy were significantly associated with health information seeking through traditional media and internet, respectively [26]. The current study is thus the first nationwide population-level study in Singapore where all citizens and residents were included in the sampling frame.

Singapore’s healthcare system is easily accessible and affordable. Care is delivered through a network of primary care clinics, with services provided by public and private hospitals and specialist centres. Primary care is delivered through 1700 general practitioners and 20 polyclinics [27], where the former provide services to meet nearly 80% of the primary care needs. There are 19 acute hospitals, 9 community hospitals and 1 psychiatric care hospital [27] that provide specialised and emergency care. Singapore has a multi-ethnic population comprising Chinese, Malay, Indian and other ethnicities, resulting in communication challenges during care delivery. Efforts have been made to cater to the needs of this diverse population by providing education materials in all four local languages (English, Chinese, Malay, and Tamil) and implementing apps that aid in communication [28].

This study examined the (a) HL of the general public, (b) factors associated with HL, and (c) association of HL with diabetes recognition, lifestyle and PA. The findings will enable health care organisations and policy makers to identify the groups that require interventions to improve HL to reduce healthcare disparities.

## 2. Materials and Methods

The methodology of this cross-sectional study, including detailed information on the sample size calculations, sampling, survey methodology and questionnaires used, was reported elsewhere [29].

### 2.1. Population

Briefly, participants were selected from a national database of Singapore residents through disproportionate sampling (ethnicity: approximately 30% each for Chinese, Malay and Indians, and 10% for other ethnic groups; and age: approximately 25% for each of the major age categories; 18 to 34 years, 35 to 49 years, 50 to 64 years, and 65 and above). This is the first nationwide survey of HL in Singapore. The sample was drawn randomly from a database containing information on all Singapore citizens and residents where each individual had an equal probability of getting selected, regardless of their geographical distribution. Disproportionate sampling ensured that the multi-ethnic population and different age groups were well-represented in the final sample and survey weights were applied, making this sample representative of the actual population. Other local studies, on the other hand, employed convenience samples from healthcare sectors and specific populations [24,25,26].

The survey was conducted face to face and offered in all four of the main languages in Singapore; English, Mandarin, Malay and Tamil. Responses were captured via computer-assisted personal interviews (CAPI). Notification letters were sent to the participants before the home visits by the trained interviewers. Eligible participants (citizens or permanent residents) included those who were 18 years and above. Those below 18 years of age, incapable of completing the study (physical or mental conditions), residing outside the country or institutionalised (e.g., in prison) during the study period were excluded. The study recruited 2895 subjects between February 2019 and September 2020, with the suspension of the study for nearly five months in response to the COVID-19 pandemic lockdown and social distancing measures (March–July 2020). The pandemic had minimal influence on the sociodemographic profile, lifestyle or physical activities of the participants, as only 0.6% of the total sample was recruited during this period. Written consent was obtained from the participants before the survey. The study followed the approved ethics procedures of the Institute of Mental Health’s Institutional Research Review Committee (IRRC) and the National Healthcare Group’s Domain Specific Review Board (NHG DSRB Ref: 2018/00430) and adhered to the declaration of Helsinki. The eligibility and response rates of the survey were 76.8% and 66.2%, respectively.

### 2.2. Sample Size

The details of the sampling, sample size and procedures of the survey were published elsewhere [29]. Briefly, the sample size was calculated for the prevalence of knowledge on diabetes and its risk factors among the general public. The prevalence of diabetes knowledge was previously estimated as 60% among the general public [30]. Calculations assumed a power of 0.8 and Type 1 error was controlled at α = 0.05. The significance was assumed at a *p* value of ≤0.05. The sample size was adjusted to account for the design effects. A sample size of 3000 was estimated to be sufficient for this study. A disproportionate stratified sampling was used based on 12 strata: four for different ethnicities (Chinese, Malay, Indian and others) and age groups (18–34 years, 35–49 years, 50–64 years and 65 years and above.

### 2.3. Questionnaire

#### 2.3.1. Sociodemographic Information

The sociodemographic information captured included age (grouped to 18–34 years, 35–49 years, 50–64 years and above 65 years), gender, ethnicity (Chinese, Malay, Indian and others), marital status (single, married, divorced/separated and widowed), education (primary or below, secondary, pre-university/junior college/vocational institute, diploma and degree and above), employment status (employed, unemployed and economically inactive), and income (no income, below SGD 2000, SGD 2000–3999, SGD 4000–5999, SGD 6000 and above).

#### 2.3.2. Brief Health Literacy Screen

The Brief Health Literacy Screen (BHLS) was developed and validated by Wallston et al. [31] and Chew et al. [32,33] in clinical cohorts. The scale has been extensively used in other populations (clinical and general population) [18,34,35], and measures the functional HL of the population.

The scale includes three items: (a) “How often do you have problems learning about your medical condition because of difficulty understanding written information?”; (b) “How often do you have someone help you read hospital materials?”, and (c) “How confident are you in filling out medical forms (e.g., forms related to health information, admissions, insurance, etc.) which were given to you by general practitioners or health providers by yourself?” (this question was modified through cognitive testing to add in the following text: ‘(e.g., forms related to health information, admissions, insurance, etc.) which are given by your general practitioners (GP) or healthcare providers by yourself?’). These questions were scored on a 5-point response option (for questions a and b: ‘all of the time, most of the time, some of the time, a little of the time, and none of the time’; for question c: ‘extremely, quite a bit, somewhat, a little, and not at all’) [31]. Scores ranging from 1 to 5 were assigned to the response options. The confidence question (item ‘c’) was reverse scored. The total score ranged from 3 to 15, with higher scores indicating higher HL. A score of ≤9 indicates inadequate HL, while a score above 9 indicates adequate HL [18,36,37]. The internal consistency of the three items was high at Cronbach’s α = 0.84.

#### 2.3.3. Diabetes Recognition

Participants were given vignettes, a short story with a hypothetical character who was matched with the gender and ethnicity of the participant. The character in the vignette was described as having typical symptoms of diabetes. The participants were asked what they thought the person in the vignette was suffering from. The responses were labelled as “correct” if they identified diabetes correctly. The vignettes were prepared in consultation with expert diabetologists and were cognitively tested to ensure that they represented the core symptoms and features of diabetes in a language understandable by the local population [38].

#### 2.3.4. Physical Activity

Physical activity (PA) among the participants was measured with the Global Physical Activity Questionnaire (GPAQ) [39,40]. The 16-item questionnaire captures the duration and frequency of physical activity (vigorous and moderate activities at work or fitness/sports activities, and travel), on a typical week. Physical activity was classified as sufficiently active (at least 150 min of moderate-intensity exercise, or 75 min of vigorous-intensity exercise, or an equivalent combination of moderate- and vigorous-intensity exercise) or insufficiently active (did not meet the aforementioned criteria) [41].

#### 2.3.5. Chronic Conditions Checklist

The questionnaire used to capture the chronic medical conditions was previously employed in population wide studies [42,43]. The questionnaire includes a list of 18 chronic conditions adapted from the World Mental Health Composite International Diagnostic Interview (CIDI) [44]. These chronic conditions, which are prevalent among the Singapore population included diabetes, asthma, cancer, arthritis, hypertension, neurological conditions, Parkinson’s disease, heart diseases, congestive heart failure, back problems, stomach ulcer, chronic inflamed bowel, kidney diseases, thyroid disorders, migraines, chronic lung conditions, stroke and hyperlipidaemia. For the regression analyses, responses were grouped into three categories: no chronic conditions, at least one chronic condition, and multimorbidity (i.e., two or more chronic conditions).

#### 2.3.6. Lifestyle Item

The lifestyle of the participants was captured through a single item “Which of the following statements best applies to you?”. The response options were (a) “I have a very healthy lifestyle”, (b) “I have a fairly healthy lifestyle”, (c) “my lifestyle can be improved” and (d) “my lifestyle is not healthy”. Response options ‘a’ and ‘b’ were grouped as “healthy lifestyle”, while ‘c’ and ‘d’ were grouped as “unhealthy lifestyle.”

### 2.4. Data Analysis

A weighted analysis of the data was performed to adjust for the oversampling, non-response and age/ethnicity distributions, with post stratification by age and ethnicity. Frequencies and weighted percentages were calculated for categorical variables. Chi-square tests were used to examine bivariate associations between each variable and HL. First, a multivariable logistic regression analysis with HL (adequate vs. inadequate) as the outcome was used to examine associations with sociodemographic correlates (i.e., age, gender, ethnicity, education, marital status, employment, income). Following that, three logistic regression models with the outcomes of diabetes recognition (correct vs. incorrect), PA (sufficiently active vs. insufficiently active), lifestyle (healthy vs. unhealthy) and HL as a predictor variable were tested. A multinomial logistic regression model with chronic conditions as the outcome (reference group of no chronic condition vs. one chronic condition or multimorbidity) and HL as a predictor was also tested. In the aforementioned logistic and multinomial logistic regression models, we adjusted for the effect of all sociodemographic variables (age, gender, ethnicity, education, marital status, employment, income). All of the analysis was performed using STATA version 15.

## 3. Results

### 3.1. Socio-Demographic Characteristics

The participants in this study had a mean age of 45.8 (±16.9) years, 51.6% were females, 75.8% were of Chinese ethnicity, 40.7% had an education status of secondary or below, 61.7% were married and 70.5% were employed (Table 1).

### 3.2. HL and Associated Sociodemographic Factors

The HL of the population was high (80.5%). Compared to individuals aged 18–34 years old, all older age groups had significantly lower odds of having adequate HL (Table 2). Compared to those of Chinese ethnicity, Malays (OR: 1.4, 95% CI:1.1–1.9, *p* = 0.02) and Indians (OR: 1.5, 95% CI:1.1–2, *p* = 0.01) had higher odds of having adequate HL. Compared to those with higher education (degree and above), those with primary school and below (OR: 0.05, 95% CI:0.0–0.1, *p* < 0.001) and secondary school education (OR: 0.2, 95% CI:0.1–0.5, *p* < 0.001) had lower odds of having adequate HL. Compared to those who are employed, unemployed adults had higher odds of adequate HL (OR: 2.6, 95% CI:1.2–5.5, *p* = 0.02). Likewise, compared to married adults, those who were divorced/separated had higher odds of adequate HL (OR: 2.5, 95% CI:1.1–5.3, *p* = 0.02).

### 3.3. Association of HL with Diabetes Recognition, Chronic Conditions, Lifestyle and PA

No significant differences in diabetes recognition were observed between the groups; 83.7% and 82.2% of those with adequate and inadequate HL, respectively, recognised diabetes correctly (Table 3). Nearly half of the sample had no chronic conditions (47.6%). A significant difference in the number of chronic conditions was noted at bivariate level between those with adequate and inadequate HL. Overall, 49.4% of those with adequate HL had no chronic conditions, compared to 32.7% in the inadequate HL group (*p* < 0.001). Similarly, only 24% of those with adequate HL had two or more chronic conditions (multimorbidity), compared to 40.9% in the inadequate HL group. Those with adequate HL showed higher levels of sufficient physical activity (84.4%) than those with inadequate HL (79.3%). However, this difference was not statistically significant. The majority of the sample indicated that they had an unhealthy lifestyle (57.4%). There were no significant differences between groups in terms of their lifestyle.

## 4. Discussion

This study is the first nationwide study in Singapore that looked at the functional HL of the local population. The results from the study showed that the current population had high functional HL (80.5%), which should enable them to function efficiently in the health care context; to read, understand and respond adequately to healthcare communications. Our finding of a high prevalence of HL is different from other population level studies. There are several explanations for this difference. A nationwide study conducted in South Korea showed that the majority of the population (61%) had inadequate HL [20], versus 19.3% in our study. A similar population-wide study in the Iranian population showed that 45.7% had inadequate HL [45], whereas a study conducted among literate Iranian adults (18–65 years old) showed that 18.1% had inadequate HL [46]. The European HL survey showed that 47% of the individuals had inadequate HL [14]. A recent study in Portuguese reported adequate HL in 92% of the sample [47]. The population characteristics of these studies were different (literate or specific age groups vs. representative population) and they measured different domains of HL (functional HL in the current study vs. critical/interactive HL). The study conducted in Korea assessed the ability (The Newest Vital Sign, 6 questions) to read and apply the information from a nutritional label as a measure of HL [20], while the Iranian study employed ‘The Health Literacy of Iranian Adults Scale’ that captured the participants’ ability to access, read, understand, appraise and decide, as five domains of HL [46], the European HL survey [14] used a scale with 47 items across 12 subdomains addressing participants’ ability to access, understand, appraise and apply information in the context of health status, health service utilisation, community participation and health behaviour. Thus, the differences in these results could be attributed to the different study methodologies including lack of common assessment tools. Apart from study specific characteristics, the economic status of the country could also play a role in the HL of the population. A systematic review of 19 articles showed that urban populations have higher HL than rural populations [48]. Another systematic review of 54 studies that compared the HL of populations between developed and developing countries concluded that those in developed countries have higher HL than in developing countries [49]. Thus, disparities in findings between various studies could also be explained based on the systematic national health policy spearheaded by the government in developed countries [50]. HL is a relatively new concept which has yet to gain traction at the policymaker level in developing countries. Singapore is an urban country with a literacy rate as high as 97.5% [51]. The country has embraced the concept of HL in the past decade and has implemented HL interventions for healthcare professionals and the general public aimed at promoting HL [50]. Higher HL of the population has been suggested to be an important outcome of health promotion campaigns in the nation [3].

Our data showed that a nearly equal proportion of individuals with both adequate and inadequate HL (57.7% vs. 56.3%) indicated having an unhealthy lifestyle. Nonetheless, our analysis did not show significant associations between HL and lifestyle, diabetes knowledge and PA. Studies have shown that HL is positively associated with lifestyle [52], PA [53] and disease knowledge [13]. A study among patients with hypertension showed that higher HL is linked to higher disease knowledge, which in turn improved the self-efficiency that promoted PA and thus, health outcomes [54]. Our findings can be explained based on the fact that although individuals have adequate HL, it does not often translate into behavioural practices [55]. The authors explained this phenomenon based on ‘patient inertia’, which is a state that involves lack of motivation to indulge in healthy behaviours, which further delays protective behaviours despite being cognisant of the benefits and consequences of such actions. A recent study that assessed the association of HL with disease knowledge reported no significant association of HL with disease knowledge of the ongoing pandemic, whereas a significant association was noted with attitudes towards prevention strategies [47]. The authors explained that while knowledge is important, it is not sufficient to bring about a positive change in behaviour. Thus, attitudes which are dependent on their beliefs, values, emotions, etc. could be a better predictor of adequate HL than knowledge, as they are likely to bring about a behaviour change.

HL was shown to be inversely associated with chronic diseases [52]. Adams and colleagues [56] showed that those with inadequate functional HL had higher chances of diabetes, stroke and heart diseases. Those with inadequate HL have poorer self-management of chronic conditions [57], low uptake of preventive services [58] and have higher mortality rate than those with adequate HL [59]. While we observed a significant group difference between those with adequate and inadequate HL in bivariate analysis, the association was lost in the adjusted multivariable analysis. The prevalence data showed a lower percentage of individuals with multimorbidity amongst those with adequate than inadequate HL. Additionally, a higher percentage of individuals with no chronic conditions was observed in the adequate HL group.

Our data showed that those belonging to the middle/older age groups, Chinese ethnicity, lower educational status (secondary and below), married, and employed were associated with inadequate HL. Similar findings were reported in other population-level studies [20,60,61]. Heijmans et al. [15] in their study among those with chronic conditions, showed that middle/older age groups, lower income and lower education status were associated with inadequate HL. Jeong and Kim [20] observed in their nationwide study that older age, lower education and geographic factors act as barriers to access, reading and understanding the health-related information even in the absence of any language barriers leading to inadequate HL. Healthcare providers should pay attention to older adults and those with lower education, to ensure that they understand the information given out to them and can make decisions with adequate awareness on the risk and benefits of their actions. Those with low HL are unlikely to engage in a discussion with care providers [62], and hence clinicians often assume that the information delivered has been comprehended by the person and an informed decision was made through a patient-centric approach. This can be overcome by incorporating HL screening into clinical care and adjusting the communication process according to the literacy level, education and age of the patient. Such approaches have been shown to improve treatment outcomes in diabetes patients [63]. Given the rapidly aging population worldwide, HL interventions should focus on these groups to reduce inequalities and health disparities.

We observed that employment was associated with inadequate HL which contradicts the results from other studies [64,65,66] where unemployment was associated with inadequate HL. Post hoc analysis of our data showed that those who were unemployed had higher odds of multimorbidity (OR:2.63, 95% CI: 1.3–5.4, *p* = 0.01), after adjusting for other sociodemographic correlates. A nationwide study delineated relationship between HL and chronic conditions showed that those with one or more chronic conditions had significantly higher likelihood of adequate HL [67]. This can be explained as a higher motivation to gain knowledge on account of the diagnosis [67] and familiarity with the health information due to frequent visits to the clinics. Karl and McDaniel [68] further confirmed this in a study of well-educated employed adults and showed a low or inadequate HL in the study sample showing that employment status does not always directly translate to adequate HL.

We also observed that Malays and Indians had higher odds of adequate HL than Chinese people. This corroborates related research which reported ethnic differences in HL [69,70,71,72,73]. Blom et al. [72] discussed the ethnic differences in HL and emphasised that language barriers and cultural factors could be possible reasons for this observation. Sentell et al. [74] studied Asian American and Pacific Islanders and showed that HL varies across different ethnic groups, with 23.9% of Filipinos showing low HL compared to 13.2% for Whites. Cunningham et al. [75] showed that while Chinese, Indian and White respondents had similar levels of HL, Chinese respondents had a lower likelihood of having adequate HL than White participants. Studies have shown that the Indian and Malay ethnicities have a higher incidence of chronic diseases and mortality compared to the Chinese [76,77,78]. Having a chronic condition is likely to improve the HL of individuals, as it exposes them to an abundance of health information from care providers and support groups [67].

It was also observed that divorced/separated individuals had higher odds of adequate HL than married individuals, an observation also noted by Liu et al. [79]. Contradicting reports showed that marriage is positively associated with HL [80,81]. We have noted that the divorced group in our study had a significantly lower proportion of young adults (12.2% vs. 8%; 21–34 years of age) and a higher proportion of older adults (36% vs. 40%, 50–64 years of age). It is plausible that this disagreement in the finding between the studies could also be due to the higher independence, competence, sense of responsibility and control that they develop post-divorce, which is triggered by the need to lead the family and take control of their life without external help, thereby facilitating a personality/lifestyle change compared to those who are married [82]. Further research is needed to gain a better understanding of the influence of marital status on HL.

The study is a nationwide survey which was conducted in four different languages (English, Chinese, Malay and Tamil) to overcome the language barrier for participation. The large sample size, randomised design and survey weighted analysis improves the generalisability of the results. The data for chronic conditions, PA and lifestyle were collected through self-report which were subjected to recall and social desirability bias. While we have analysed HL and associated factors in this study, patient activation, self-care and management are important factors that determine health outcomes which were not assessed. Additionally, the cross-sectional study design does not allow analysis of the causal relationship between the variables which is a limitation of the study. The BHLS is not validated in the local population which is a limitation of the study. Nonetheless, cognitive testing of the scale was performed to ensure that it is suitable for the local population. Apart from these, the HL of the family members and the social network of the individual that could influence the health outcomes were not captured in this study.

This study has key clinical implications. Currently, HL assessments are not routinely performed in healthcare settings, and the HL status of patients is unknown. In multi-ethnic/multilingual populations, language barriers could further complicate the process. Routine HL assessments which are quick and cost-effective should be incorporated in clinic visits, and communication strategies and educational materials should be modified accordingly. Patients might have difficulty comprehending the medical information conveyed to them by the healthcare team and may often feel uncomfortable to ask for clarification. This leads to communication failures [83]. Older adults and those with lower education who tend to have low HL are unlikely to use sophisticated platforms for accessing health-related information; thus, materials that are user-friendly and user-involved are needed to improve their HL. Health care teams need to be trained to fill in the HL deficit and unmet needs of these socio-demographic groups to avoid health inequalities.

## 5. Conclusions

The study provided a national-level prevalence of HL in Singapore. The results show that the majority of the population had adequate HL. Older adults, those of Chinese ethnicity, those who were married, those who were employed and those with lower levels of education had higher odds of inadequate HL. Given that inadequate HL is generally associated with adverse health outcomes, the socio-demographic groups who are unlikely to have sufficient HL should be identified, and suitable interventions should be undertaken to ameliorate health disparities.

## Figures and Tables

**Table 1:** Sociodemographic characteristics of the population.

	Overall (n = 2895)		Adequate HL (>9) (n = 2332)		Inadequate HL (≤9) (n = 558)	
	n	% ^a^	n ^b^	% ^c^	n	% ^c^
Age group						
21–34	823	29.9	770	94.7	52	5.2
35–49	719	28.2	654	87.9	65	12.1
50–64	774	26.8	574	70.5	198	29.4
65 and above	579	15.1	334	56.3	243	42.7
Gender						
Female	1474	51.6	1141	78.4	331	21.4
Male	1421	48.5	1191	82.7	227	17.1
Ethnicity						
Chinese	796	75.8	623	79.1	171	20.7
Malay	974	12.7	760	82.0	211	17.7
Indian	918	8.6	756	86.9	162	13.1
Others	207	2.9	193	92.9	14	7.1
Education						
Primary and below	637	20.4	276	42.0	359	57.2
Secondary School	684	20.3	575	79.4	107	20.5
Pre-U/Junior College	126	4.8	116	96.1	10	3.9
Vocational Institute/ITE	267	6.6	243	88.5	24	11.5
Diploma	479	18.5%	446	92.3	32	7.7
Degree and above	702	29.5	676	96.2	26	3.8
Marital status						
Single	731	29.2	661	91.2	69	8.7
Married/cohabiting	1860	61.7	1479	77.0	377	22.7
Divorced/separated	154	5.0	122	80.3	32	19.7
Widowed	149	4.1	69	56.6	80	43.4
Refused	1	0.0	1	100	0	0.0
Employment						
Employed	1933	70.5	1637	83.2	293	16.7
Economically inactive	829	25.4	582	72.3	245	27.3
Unemployed	133	4.1	113	84.9	20	15.1
Monthly Personal Income (in SGD)						
Below 2000	1236	38.3	849	68.5	384	31.3
2000 to 3999	698	23.9	607	84.8	90	15.1
4000 to 5999	318	12.8	300	92.1	18	7.9
6000 and above	300	13.5	290	93.8	10	6.2
No income	219	7.0	169	74.4	49	24.4
DK/RF	124	4.5	117	96.9	7	3.1

^a^ Column percentage; ^b^ Total does not tally to 2895 due to missing data (n = 5); ^c^ Row percentage; SGD: Singapore Dollars.

**Table 2:** Sociodemographic correlates of adequate HL.

Variable	OR	*p* Value	95% CI	
			Lower	Upper
Age groups				
18 to 34 (Ref)				
35 to 49	0.4	0.01	0.2	0.8
50 to 64	0.3	0.003	0.2	0.7
65 and above	0.3	0.001	0.1	0.6
Gender				
Female (Ref)				
Male	1.2	0.35	0.8	1.7
Ethnicity				
Chinese (Ref)				
Malay	1.4	0.02	1.1	1.9
Indian	1.5	0.01	1.1	2.0
Others	1.3	0.49	0.6	2.6
Education				
Primary and below	0.05	<0.001	0.0	0.1
Secondary	0.2	<0.001	0.1	0.5
A-Level/Vocational School/ITE	0.5	0.09	0.2	1.1
Polytechnic	0.5	0.14	0.2	1.2
Degree and above (Ref)				
Marital status				
Single	0.9	0.72	0.5	1.7
Married/Cohabiting (Ref)				
Divorced/separated	2.5	0.02	1.1	5.3
Widowed	1.0	0.90	0.6	1.9
Employment				
Employed (Ref)				
Economically Inactive	1.4	0.14	0.9	2.2
Unemployed	2.6	0.02	1.2	5.5
Monthly income (SGD)				
Below 2000 (Ref)				
2000 to 3999	1.3	0.25	0.8	2.1
4000 to 5999	1.5	0.33	0.7	3.2
6000 and above	1.6	0.32	0.6	4.2
No income	0.7	0.18	0.4	1.2

O.R. > 1 indicates higher odds of having adequate health literacy. SGD: Singapore dollars.

**Table 3:** Prevalence of chronic conditions, diabetes recognition, healthy lifestyle and PA.

	Overall		Adequate HL (>9)		Inadequate HL (≤9)		*p* Value
	n	% ^a^	n	%	n	%	
**Chronic conditions (Including diabetes)**							
None	1243	47.6	1076	49.4	164	32.7	<0.001
At least one	760	27.3	624	26.5	135	25.4	
Two or more	884	24.9	629	24.0	255	40.9	
Missing	8	0.3	3	0.1	4	1.0	
**Diabetes Recognition**							
Correct Recognition	2441	83.5	1985	83.7	452	82.2	0.55
Incorrect Recognition (includes do not know)	454	16.5	347	16.3	106	17.8	
**Physical Activity (PA)**							
Insufficiently Active	464	16.6	329	15.6	134	20.6	0.06
Sufficiently Active	2429	83.3	2002	84.4	423	79.3	
Excluded	2	0.0	1	0.0	1	0.1	
**Lifestyle**							
Healthy	1370	42.5	1086	42.3	282	43.3	
unhealthy	1523	57.4	1246	57.7	275	56.3	0.74
Missing	2	0.1	0	0.0	1	0.4	

^a^ Column percentage. Diabetes recognition, PA, lifestyle and number of chronic conditions showed no significant association with HL. (Table 4).

**Table 4:** Association of HL with chronic conditions, diabetes recognition, PA and lifestyle.

	O.R.	*p* Value	95% CI	
			Lower	Upper
**Diabetes recognition ^a^**				
Inadequate HL ^b^ (*Ref*)				
Adequate HL	1.1	0.67	0.7	1.8
**Physical activity ^a^**				
Inadequate HL *(Ref)*				
Adequate HL	1.1	0.78	0.7	1.6
**Lifestyle ^a^**				
Inadequate HL *(Ref)*				
Adequate HL	1.3	0.10	0.9	1.9
**Chronic conditions ^c^**				
*No chronic conditions (Ref)*				
*One chronic conditions* vs. *Ref*				
Inadequate HL				
Adequate HL	0.9	0.60	0.6	1.4
*Two or more chronic conditions* vs. *Ref*				
Inadequate HL				
Adequate HL	0.8	0.36	0.6	1.2

**^a^** Logistic regression model, adjusted for sociodemographic variables. ^b^ Inadequate health literacy ≤ 9; adequate health literacy >9. **^c^** Multinomial logistic regression model, adjusted for sociodemographic variables. Reference group: No chronic conditions.

## Data Availability

The data presented in this study are available on request from the corresponding author. The data are not publicly available due to institutional policies.
